# KLHDC10 Deficiency Protects Mice against TNFα-Induced Systemic Inflammation

**DOI:** 10.1371/journal.pone.0163118

**Published:** 2016-09-15

**Authors:** Namiko Yamaguchi, Shiori Sekine, Isao Naguro, Yusuke Sekine, Hidenori Ichijo

**Affiliations:** 1 Laboratory of Cell Signaling, Graduate School of Pharmaceutical Sciences, The University of Tokyo, Tokyo, Japan; 2 Cambridge Institute for Medical Research, University of Cambridge, Cambridge, United Kingdom; Toho Daigaku, JAPAN

## Abstract

Systemic inflammatory response syndrome (SIRS) is a form of fatal acute inflammation for which there is no effective treatment. Here, we revealed that the ablation of Kelch domain containing 10 (KLHDC10), which we had originally identified as an activator of Apoptosis Signal-regulating Kinase 1 (ASK1), protects mice against TNFα-induced SIRS. The disease development of SIRS is mainly divided into two stages. The early stage is characterized by TNFα-induced systemic necroptosis, a regulated form of necrosis mediated by Receptor-interacting protein (RIP) 1/3 kinases. The later stage presents with an over-production of inflammatory cytokines induced by damage-associated molecular patterns (DAMPs), which are immunogenic cellular contents released from cells that underwent necroptosis. Analysis of TNFα-challenged mice revealed that KLHDC10-deficient mice show a reduction in the inflammatory response, but not in early systemic necroptosis. *In vitro* analysis suggested that the reduced inflammatory response observed in KLHDC10-deficient mice might be caused, in part, by enhanced necroptosis of inflammatory cells encountering DAMPs. Interestingly, the enhancement of necroptosis induced by KLHDC10 deficiency was selectively observed in inflammatory cells. Our results suggest that KLHDC10 is a cell-type specific regulator of necroptosis that ultimately contributes to the development of TNFα-induced SIRS.

## Introduction

Kelch domain containing 10 (KLHDC10) was initially identified as an activator of Apoptosis Signal-regulating Kinase 1 (ASK1), a stress responsive MAP3K, through the *Drosophila* misexpression screen [1]. Recently, several lines of evidence have suggested that a large portion of the kelch repeat proteins interact with the Cullin-RING ubiquitin ligases (CRLs) and serve as substrate recognition subunits of the CRL complex [2,3,4]. KLHDC10 contains consensus sequences in its C-terminus, which are called the Cul2-box and the BC-box and are required for binding to CRL2 complex components. These features strongly support the possibility that KLHDC10 functions as a substrate receptor for the CRL2 complex [1]. Further, we previously reported that KLHDC10-dependent ASK1 activation does not rely on its putative function as a substrate receptor of the CRL2 complex but on its suppressive engagement of protein phosphatase 5 (PP5), a negative regulator of ASK1 [5]. KLHDC10 binds to the phosphatase domain of PP5 and suppresses its phosphatase activity, which ultimately contributes to oxidative stress-induced sustained activation of ASK1 and cell death [1].

TNFα (Tumor necrosis factor α) is a pleiotropic inflammatory cytokine that plays important roles in cell survival, cell death, and inflammation. Recently, it has been reported that TNFα can induce a regulated form of necrosis, which is called necroptosis, by activating receptor-interacting protein 1 (RIP1) and RIP3 [6,7]. It has been suggested that RIP1/3 kinases induce necroptosis signaling through phosphorylation of Mixed lineage kinase domain-like (MLKL), which functions as a pseudokinase [8,9,10], eventually leading to an influx of Na^+^ or Ca^2+^, depending on the cell type [11,12]. Furthermore, recent studies have indicated that reactive oxygen species (ROS) are also involved in necroptosis [10,13,14]. In particular, NADPH oxidase has been suggested as one of the main sources of ROS production [14,15,16,17].

TNFα-induced systemic inflammatory response syndrome (SIRS) is a systemic inflammation model mimicking acute inflammation caused by surgeries, bacterial infections, pancreatitis, and traumas in human patients [18]. Experimentally, SIRS is induced in mice by injecting them with an overdose of TNFα [19,20]. The pathogenesis of TNFα-induced SIRS is known to develop through two steps. The first step is systemic necroptosis, which is mediated by RIP1/3 kinases [21,22]. Because necroptotic cell death is followed by membrane rupture, cells dying via necroptosis release inflammatory cellular contents, including so-called damage-associated molecular patterns (DAMPs). The over-production of inflammatory cytokines, such as Interleukin (IL)-1β and IL-6, by inflammatory cells responding to DAMPs is the second step of SIRS development, which induces severe tissue damage [21]. The components involved in these two steps are critical determinants of lethality. In particular, inhibiting systemic necroptosis through the ablation of RIP3, or suppressing the subsequent inflammatory responses by treatment with neutralizing antibodies for specific inflammatory cytokines, confers resistance against TNFα-induced SIRS in mice [21,23,24].

Here, we showed that KLHDC10 deficiency protects mice from mortality and hypothermia in TNFα-induced SIRS. KLHDC10 deficiency did not affect early systemic necroptosis, while it diminished the subsequent inflammatory responses, including IL-6 production, possibly by promoting the necroptosis of inflammatory cells under exposure to DAMPs. Our study may provide a clue to the identification of potential therapeutic targets for SIRS.

## Materials and Methods

### Cell culture

RAW264.7 cells (ATCC), immortalized mouse embryonic fibroblasts (MEF), and L929 cells (gited by Dr. Uojima, Niigata Univ.) were maintained in Roswell Park Memorial Institute (RPMI)-1640, Dulbecco’s modified Eagle medium (DMEM) containing 4.5 mg/ml glucose, and DMEM containing 1.5 mg/ml glucose respectively (Sigma). The culture media were supplemented with fetal bovine serum (FBS, Biowest) and 100 units/ml penicillin (Meiji Seika Pharma) and maintained under 5% CO_2_ at 37°C. The methods used for MEF dissection were described previously [25]. Cells were then immortalized by transfection with Simian vacuolating virus 40 (SV40) large T antigen.

### Reagents

Recombinant mouse TNFα (mTNFα) was produced in *E*. *coli* and purified as previously described [26]. Endotoxins were removed with ToxinEraser^TM^ Endotoxin Removal Resin (GenScript). Smac-mimetic (LCL-161, Active Biochem), Z-VAD-fmk (Sigma), and Necrostatin-1 (Sigma) were dissolved in Dimethyl suloxide (DMSO, Sigma) and diluted in culture medium.

### siRNAs and transfection

RAW264.7 cells, immortalized MEF cells, and L929 cells were transfected with the following siRNA oligonucleotides using the Lipofectamine RNAiMAX reagent (Invitrogen) according to the manufacturer’s instructions: for mouse KLHDC10 #1, AUGAGUUCCUGUGUGAGUCCAAGGU; for mouse KLHDC10 #2, AAGACAUAAAGGGACCCAUUGAUGA; and for mouse KLHDC10 #3, UUUCAUGUCUGUACCUCUCCUCCGG. Stealth RNAi^TM^ Negative Control Medium GC Duplex and Stealth RNAi^TM^ Negative Control Medium GC Duplex #3 were used as controls. siRNAs were used at a final concentration of 40 nM (RAW264.7 cells) or 20 nM (immortalized MEF cells and L929 cells). After 48 hours, knockdown was analyzed by immunoblotting with the indicated antibodies.

### Cell death assay

Smac-mimetic and Z-VAD-fmk-induced cell death was monitored using a LDH-Cytotoxic Test Wako (WAKO) according to the manufacturer’s protocol. The activity of LDH released into the culture medium was quantified as a percentage of the total LDH activity using Labsystems Multiskan^®^ Biochromatic (Labsystems).

### Quantitative PCR

Total RNA was isolated from each organ or RAW264.7 cells using Isogen (Wako) and reverse-transcribed to cDNA using ReverTra Ace^®^ qPCR RT Master Mix with gDNA Remover (Toyobo). Quantitative PCR was performed with a LightCycler^®^ system (Roche). The sequences of the primers used were as follows: mouse IL-6, 5’-GCTACCAAACTGGATATAATCAGGA-3’ (sense) and 5’-CAGTGATGGCGAAGGCTATT-3’ (antisense); mouse 40S ribosomal subunit protein S18 5’-TCCAGCACATTTTGCGAGTA-3’ (sense) and 5’-CAGTGATGGCGAAGGCTATT-3’ (antisense). All mRNA expression levels were normalized to S18 expression.

### Antibodies

For immunoblotting analysis, antibodies against actin (Sigma), Nox2 (gp91phox, BD Transduction Laboratories^TM^), and α-tubulin (Serotec) were purchased. The rat monoclonal anti-KLHDC10 antibody (S-1) was generated as previously described [1].

### Immunoblotting analysis

Cells were lysed on ice in IP lysis buffer (20 mM Tris-HCl, pH7.5, 150 mM NaCl, 10 mM EDTA, pH7.5, 1% sodium deoxycholate, and 1% Triton X-100) with protease inhibitors (1 mM phenylmethylsulfonyl fluoride, and 5 μg/ml leupeptin). After centrifugation, cell extracts were resolved on SDS-PAGE and electroblotted onto polyvinylidene difluoride membranes. After blocking with 5% skim milk in TBS-T (50 mM Tris-HCl, pH8.0, 150 mM NaCl, and 0.05% Tween 20), the membranes were probed with antibodies against KLHDC10 or actin. The proteins were detected with an ECL system (GE Healthcare).

### Mice

C57BL/6J mice were obtained from CLEA Japan, Inc. (Tokyo, Japan). Mice were housed in a specific pathogen-free animal facility. All mice were housed at a temperature of 25°C under a 12 hour-dark/light cycle and fed with γ-ray irradiated normal diet (ORIENTAL YEAST). Mice were checked at least five days a week and cared as necessary. Mating was done in separate cages from other mice and each pregnant mouse was housed in different cages in order to secure the appropriate environment for child rearing. All mouse experiments were performed in accordance with protocols approved by the Animal Research Committee of the Graduate School of Pharmaceutical Sciences at the University of Tokyo (Tokyo, Japan).

### Generation of KLHDC10 KO mice

A targeting vector was constructed to replace the first exon (Exon 1) of the *KLHDC10* gene, containing a start codon with a reverse-oriented PGKp-neo^r^ cassette. An upstream fragment that was 4.7 kb and a downstream fragment that contained 3.8 kb of Exon 1 were used as homologous regions for recombination. The NotI-linearized targeting vector was electroporated into RENKA embryonic stem cells (Transgenic Inc.) derived from C57BL/6 mice and subjected to selection with G418. G418-resistant ES clones that contained the intended recombination were screened by Southern blot analysis. Southern blot analysis was performed by digesting genomic DNA with ScaI or BamH, and hybridizing with a specific probe, as shown in S1A Fig. Heterozygous mutant ES cells were injected into blastocysts. Germline transmission of mutated alleles to F1 mice, obtained by intercrossing the resultant male chimeras with female C57BL/6J mice, was confirmed by Southern blot analysis. Heterozygous mutant mice were back-crossed to the C57BL/6J mice, which were bred in our specific pathogen-free facility for 7 generations. Homozygous mutant mice were obtained by intercrossing heterozygous mutant mice. The ASK1 (MAP3K5)-/- mouse model has been previously described [25].

### Injections, monitoring, and sampling

Nine- to fourteen-week-old male mice were exported from the SPF facility just before the experiment. Injection, monitoring, and sampling were performed in a non-SPF facility. mTNFα (5 μg/mouse) was diluted in sterilized PBS and injected intravenously in a total volume of 200 μl. Rectal body temperature was recorded with the Multipurpose Microprobe Thermometer BAT-12 (Muromachi Kikai). Serum samples and tissue samples of spleen, liver, and small intestine were collected at the designated times after injection. Blood was obtained by cardiac puncture. We used humane endpoints during the survival study, and also tried to minimize mice suffering from distress by administering inhalant anaesthetics to mice. In detail, all animals were monitored every 2 hours for signs of distress and endpoints including hypothermia and decrease in motion. If animals meet above criteria and were enable to evade handling, they were humanely euthanized via inhaled anesthetic. All the anesthesia and euthanasia were performed in accordance with protocols approved by the Animal Research Committee of the Graduate School of Pharmaceutical Sciences at the University of Tokyo (Tokyo, Japan).

### DAMPs and cytokine bioassay

Measurements of serum ALT and LDH were performed by SRL, Inc. For the quantification of mitochondrial DNA (mtDNA), DNA was extracted from serum using a QIAamp DNA Blood mini kit (QIAGEN), and the mtDNA amounts were determined by quantitative PCR as described above. Primers used were as follows: mouse mitochondrial cytochrome B, 5’-CTTAGCCATACACTACACATCAG-3’ (sense) and 5’-ATCCATAATATAAGCCTCGTCC-3’ (antisense); mouse mitochondrial cytochrome C oxidase III, 5’-ACGAAACCACATAAATCAAGCC-3’ (sense) and 5’-TAGCCATGAAGAATGTAGAACC-3’ (antisense); mouse mitochondrial NADH dehydrogenase, 5’-AGCCTCAAACTCCAAATACTC-3’ (sense) and 5’-CCCTGATACTAATTCTGATTCTCC-3’ (antisense). The IL-1β and IL-6 serum levels were quantified using a Quantikine ELISA kit (R&D Systems). Measurement of the absorbance was performed using a Labsystems Multiskan^®^ Bichromatic (Labsystems).

## Results

### KLHDC10 knockout (KO) mice are protected against TNFα-induced SIRS

**Fig 1:**
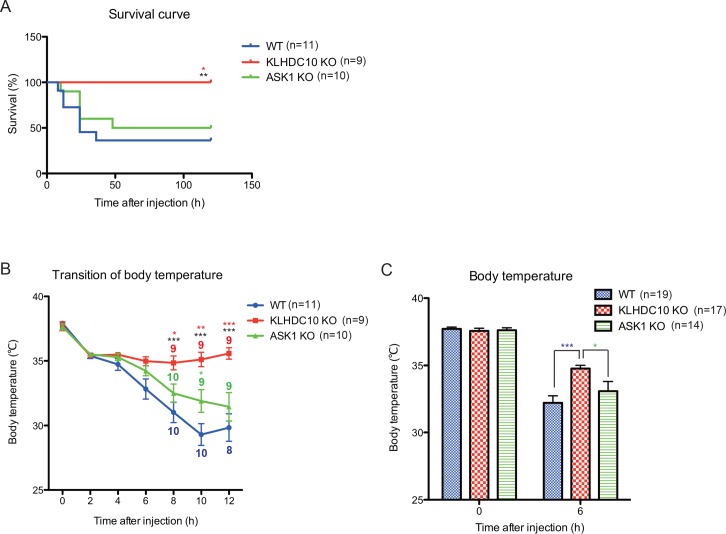
KLHDC10 knockout (KO) mice are resistant against TNFα-induced SIRS. (A) Survival period of WT, KLHDC10 KO, and ASK1 KO mice intravenously injected with 5 μg of mTNFα. (B) Body temperature of WT, KLHDC10 KO, and ASK1 KO mice intravenously injected with 5 μg of mTNFα. Each dot indicates the number of surviving mice at that time point. (C) Body temperature of WT, KLHDC10 KO, and ASK1 KO mice after 0 and 6 hours from the intravenous injection of 5 μg of mTNFα. Data are represented as the mean ± SEM. **P<0.01 versus WT mice and *P<0.05 versus ASK1 KO mice analyzed by Log-rank test followed with Gehan-Breslow-Wilcoxon test (A). ***P<0.001 (blank line) versus WT mice and *P<0.05, **P<0.01, ***P<0.001 (red line) versus ASK1 KO mice. *P<0.05 (green line) versus WT mice. All samples were analyzed by two-way ANOVA with the Bonferroni post-hoc test (B). ***P<0.001 (blue line) versus WT mice and *P<0.05 (green line) versus ASK1 KO mice analyzed by the Student’s *t* test (C).

To clarify the pathophysiological functions of KLHDC10 *in vivo*, we engineered a targeting vector to generate whole-body KLHDC10 knockout (KO) mice (S1A Fig). Integration of the KO allele was confirmed by Southern blotting (S1B Fig). The ablation of KLHDC10 protein was also confirmed by immunoblotting analysis using primary mouse embryonic fibroblasts (MEF) derived from KLHDC10 KO mice (S1C Fig). KLHDC10 KO mice were viable and did not show any developmental or homeostatic abnormalities. Thus, we examined their phenotype by testing several pathophysiological models *in vivo*. We focused on oxidative stress-related models because we previously elucidated that KLHDC10 regulates oxidative stress-induced cellular death [1]. KLHDC10-deficient mice showed an interesting phenotype in a TNFα-induced SIRS model, in which mice were challenged with high doses of TNFα. We examined the survival period and hypothermia after TNFα injection, both of which are typical parameters of TNFα-induced SIRS. As a result, we found that KLHDC10 KO mice were dramatically resistant to TNFα-induced SIRS (Fig 1A) and maintained their body temperature more efficiently than WT mice (Fig 1B and 1C). In contrast, ASK1 KO mice were not as protected from TNFα-dependent lethality and hypothermia as KLHDC10 KO mice (Fig 1A to 1C). These results suggest that KLHDC10 is required for TNFα-induced SIRS development through an ASK1-independent mechanism.

### TNFα-induced necroptosis was not systemically affected in KLHDC10 KO mice

According to previous reports, the first step of TNFα-induced SIRS is a systemic necroptosis, a type of cell death followed, by cell membrane rupture and leakage of cellular contents, including DAMPs [21,27]. Because excessive inflammatory cytokines produced at the second stage can also induce further cell death by acting on the remaining living cells, TNFα-induced SIRS develops through a vicious cycle, in which the two processes described above occur repeatedly. Eventually, mice die due to severe tissue injury, respiratory failure, sharp declines in body temperature and in blood pressure [24,28,29,30].

**Fig 2:**
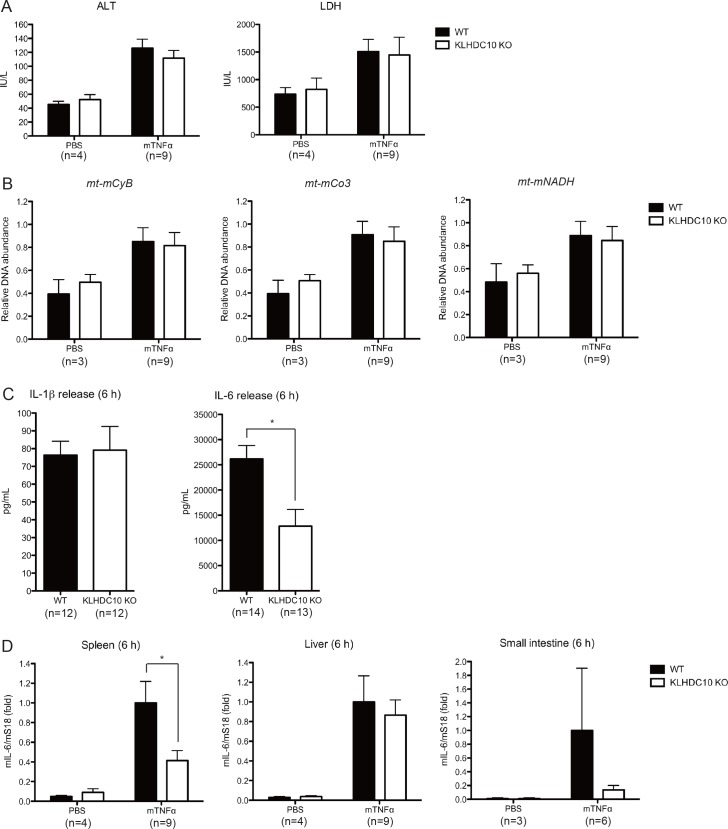
KLHDC10 deficiency does not reduce systemic necroptosis but impairs the inflammatory response in TNFα-induced SIRS. (A) Serum samples of WT and KLHDC10 KO mice were collected at 6 hours after challenge with 5 μg of mTNFα or PBS, followed by analysis for alanine aminotransferase (ALT) and lactate dehydrogenase (LDH). (B) Relative DNA abundance in sera derived from WT and KLHDC10 KO mice at 6 hours after challenge with 5 μg of mTNFα or PBS was analyzed by quantitative real-time PCR for *mitochondrial mouse Cytochrome B (mt-mCyB)*, *Cytochrome C oxidase III (mt-Co3)*, and *NADH oxidase (mt-NADH)*. (C) Serum samples of KLHDC10 KO and WT mice were collected at 6 hours after challenge with 5 μg of mTNFα, followed by analysis for interleukin-1β (IL-1β) and IL-6 by ELISA. (D) Six hours after challenge with 5 μg of mTNFα or PBS, *IL-6* gene expression was determined by quantitative real-time PCR. *S18* gene expression level was examined as an internal control for normalization. Data are represented as the mean ± SEM. *P<0.05 versus WT mice analyzed by the Student’s *t* test.

Based on these studies, we examined the involvement of KLHDC10 in the two stages described above. To address the possibility that KLHDC10 deficiency may affect systemic necroptosis, we examined several tissue damage markers in the serum after TNFα injection. Previous reports suggest that RIP3 deficiency or Necrostatin-1 (a RIP1 kinase inhibitor [31]) pretreatment significantly suppress tissue damage markers, including ALT, LDH, and mitochondrial DNA, protecting mice from mortality and hypothermia in TNFα-induced SIRS [21]. The TNFα–induced increase in serum ALT and LDH at 6 hours after injection was almost comparable between WT and KLHDC10 KO mice (Fig 2A). We also quantified the expression of three mitochondrial genes, *Cytochrome B (CyB)*, *Cytochrome C oxidase III (Co3)*, and *NADH dehydrogenase (NADH)*, released into the serum. Although the expression of all these genes was increased at 6 hours after TNFα injection, as reported in a previous study [21], no difference was observed between WT and KLHDC10 KO mice (Fig 2B). These results suggest that KLHDC10 deficiency does not affect TNFα-induced systemic necroptosis, an important first step for disease development of SIRS.

### The production of the inflammatory cytokine IL-6 after TNFα injection was reduced in the serum of KLHDC10 KO mice

A previous report suggests that the sustained cytokine increase at later time points, such as 6 hours after TNFα injection, is an indirect response of DAMPs release, rather than a direct consequence of TNFR signaling [21]. Thus, we examined the serum concentrations of IL-1β and IL-6 at 6 hours after TNFα injection by ELISA (Fig 2C). Although the production of IL-1β in KLHDC10 KO mice was almost comparable to that of WT mice (Fig 2C, left panel), the serum concentration of IL-6 in KLHDC10 KO mice was significantly lower than in WT mice at 6 hours after TNFα injection (Fig 2C, left panel). Our findings suggest that KLHDC10 deficiency affects the second step of SIRS development, the production of DAMPs-dependent inflammatory cytokines.

### IL-6 production in the spleen was reduced by *KLHDC1*0 deletion in TNFα-induced SIRS

We next examined the expression levels of IL-6 mRNA in several tissues. As shown in Fig 2D, IL-6 mRNA expression was induced in the spleen, liver, and small intestine at 6 hours after TNFα injection. Among these tissues, IL-6 mRNA levels were significantly decreased in KLHDC10 KO mice, especially in the spleen at 6 hours after TNFα injection (Fig 2D, left panel). A similar pattern was observed in the small intestine of KLHDC10 KO mice (Fig 2D, right panel). By contrast, IL-6 mRNA expression was not diminished in the liver of KLHDC10 KO mice (Fig 2D, middle panel). These results suggest that KLHDC10 deficiency affects the DAMPs-dependent inflammatory response in SIRS development in a tissue-dependent manner.

### DAMPs-induced IL-6 production in KLHDC10-deficient inflammatory cells is not reduced *in vitro*

**Fig 3:**
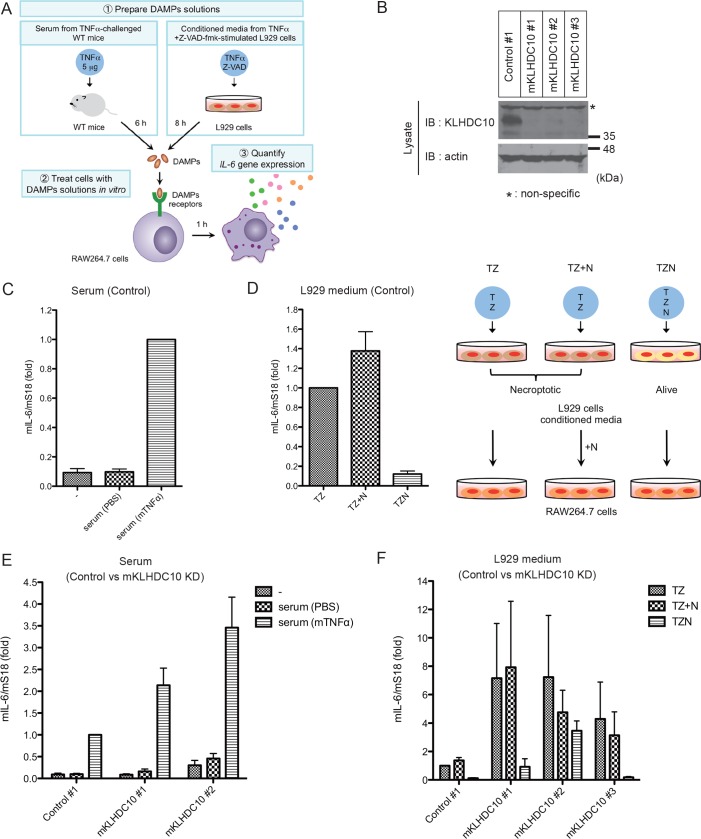
KLHDC10 deficiency does not reduce the production of DAMPs-induced inflammatory cytokines in inflammatory cells *in vitro*. (A) Schematic abstract of the experimental procedure. (B) The knockdown efficiency of KLHDC10 was determined by immunoblotting analysis after transfection of RAW264.7 cells with the indicated siRNAs. (C, D) RAW264.7 cells transfected with control siRNAs were stimulated with the indicated mouse sera or L929 cell conditioned media. After 1 hour, *IL-6* gene expression in the indicated tissues was determined by quantitative real-time PCR (n = 3). (E, F) RAW264.7 cells transfected with control or mKLHDC10 siRNAs were stimulated with the indicated mouse sera or L929 cell conditioned media. After 1 hour, *IL-6* gene expression in the indicated tissues was determined by quantitative real-time PCR (n = 3). Data are represented as the mean ± SEM. T: mTNFα (20 ng/ml), Z: Z-VAD-fmk (10 μM), N: Necrostatin-1 (10 μM). TZ+N indicates TZ-stimulated cultured medium plus Necrostatin-1.

Next, we pursued the identification of the factors mediating the reduction of DAMPs-dependent inflammatory cytokine IL-6 in KLHDC10 KO mice. The spleen is abundant in inflammatory cells, such as macrophages. Therefore, we focused on the response of inflammatory cells against DAMPs. Not only proteins but also other molecules, including DNA and lipids, can act as DAMPs [32,33]. Thus, to prepare a mixture of DAMPs, we collected serum from WT mice at 6 hours after TNFα injection (Fig 3A, left panel). In addition, we harvested the conditioned media of L929 cells that underwent necroptosis (Fig 3A, right panel). The conditioned medium was collected after stimulating cells with TNFα and Z-VAD-fmk, a pan-caspase inhibitor, for 8 hours, when the majority of the cells had died and various DAMPs had been released into the media [34,35]. As recipient cells that were subjected to each DAMPs solution, we used the macrophage cell line RAW264.7 as a model of inflammatory cells. As shown in Fig 3C and 3D, both of the DAMPs solutions strongly induced IL-6 mRNA expression in RAW264.7 cells. Moreover, when the induction of necroptosis in L929 cells was inhibited by the presence of Necrostatin-1, a RIP1 kinase inhibitor, treatment with conditioned media from L929 cells did not induce IL-6 mRNA expression in RAW264.7 cells (Fig 3D, bar filled with horizontal stripes), indicating that we successfully reproduced the regulation of IL-6 production induced by DAMPs, which was released from necroptotic L929 cells. By using this *in vitro* evaluation system, we examined the effects of KLHDC10 knockdown on DAMPs-induced IL-6 mRNA expression. We confirmed that the KLHDC10 protein expression levels were suppressed upon siRNA transfection of RAW264.7 cells (Fig 3B). Unexpectedly, the induction of IL-6 mRNA expression observed in RAW264.7 cells was not reduced but was enhanced in KLHDC10 knockdown cells when any of the previously described DAMPs solutions was utilized for stimulation (Fig 3E and 3F). These results indicate that DAMPs-induced IL-6 production per cell is not reduced in KLHDC10-deficient inflammatory cells.

### KLHDC10 deficiency in inflammatory cells promotes DAMPs-induced cell death

**Fig 4:**
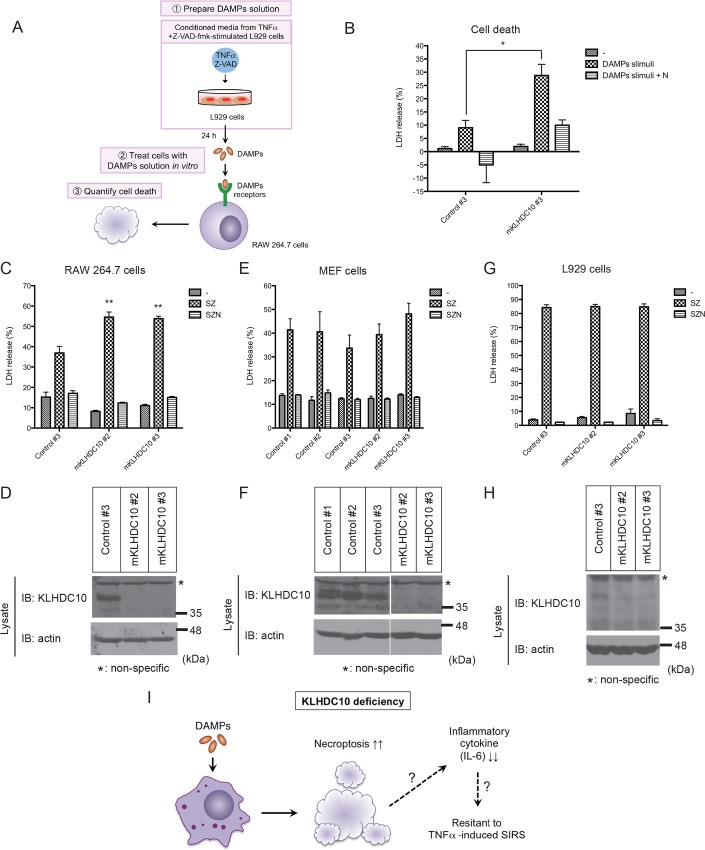
KLHDC10 deficiency selectively enhances necroptosis in inflammatory cells *in vitro*. (A) Schematic abstract of the experimental procedure. (B) RAW264.7 cells transfected with control or mKLHDC10 siRNAs were stimulated with the indicated L929 cell conditioned media. After 24 hours, LDH release was quantified as an indicator of cell death (n = 3). (C, E, G) RAW264.7 cells (C), immortalized MEF cells (E), and L929 cells (G) transfected with control or mKLHDC10 siRNAs were stimulated as indicated. After 24 hours, LDH release was quantified as an indicator of cell death (n = 3 each). (D, F, H) In RAW264.7 cells (C), immortalized MEF cells (E), and L929 cells (G), the knockdown efficiencies of KLHDC10 were determined by immunoblotting analysis after transfection with the indicated siRNAs. (I) Summary of this study (see the detailed explanation in the text). Data are represented as the mean ± SEM. *P<0.05 versus control siRNA analyzed by Student’s *t* test (B). **P<0.01 versus control siRNA analyzed by one-way ANOVA with Dunnette’s post-hoc test (C). T: mTNFα (20 ng/ml), S: Smac-mimetic (50 nM), Z: Z-VAD-fmk (10 μM), N: Necrostatin-1 (10 μM).

To investigate the lack of DAMPs-induced IL-6 production in KLHDC10-deficient spleen, we hypothesized that KLHDC10 deficiency may cause a decrease in the total number of inflammatory cells in response to DAMPs, which ultimately results in decreased IL-6 production. Using a similar method to Fig 3, we prepared DAMPs-containing conditioned media of necroptotic L929 cells and incubated RAW264.7 cells with the conditioned media for 24 hours (Fig 4A). LDH release was quantified as an indicator of cell death. LDH was quantified by subtracting the initial LDH contents in the conditioned media from those in each sample. As shown in Fig 4B, DAMPs-induced cell death was significantly enhanced in KLHDC10-deficient RAW264.7 cells. We could not detect cell death when RAW264.7 cells were treated with TNFα and Z-VAD-fmk alone (S2A Fig). This observation confirms that the cell death detected after incubation with the conditioned media was mediated not by residual TNFα-Z-VAD-fmk but by DAMPs. In addition, the cell death detected after incubation with the conditioned media was inhibited by Necrostatin-1, suggesting that RIP1/3-dependent necroptosis may be involved in DAMPs-induced cell death (Fig 4B). These results suggest that KLHDC10 deficiency in inflammatory cells promotes RIP1/3-dependent necroptosis in response to DAMPs.

### KLHDC10 deficiency selectively promotes RIP1/3 kinase-dependent cell death in inflammatory cells

While necroptosis is induced through the activation of RIP1/3 kinases, various other stress responses, such as survival, inflammation, and apoptosis, are also induced downstream of RIP1. Co-treatment with Smac-mimetic, an IAP inhibitor [7,36], and Z-VAD-fmk has been reported to evoke necroptosis. Smac-mimetic inhibits the survival signal through the NF-ĸB pathway, while Z-VAD-fmk suppresses apoptosis by inhibiting caspase activity. In summary, treatment with Smac-mimetic plus Z-VAD-fmk causes necroptosis by suppressing the signaling pathways of survival and apoptosis. Henceforth, we will indicate the co-treatment of Smac-mimetic and Z-VAD-fmk as “SZ stimuli”. RIP1/3 are involved in enhancing DAMPs-induced cell death in KLHDC10-deficient RAW264.7 cells. Thus, we next examined whether RIP1/3-dependent necroptosis was also enhanced by KLHDC10 deficiency in RAW264.7 cells after treatment with SZ stimuli. Cell death was quantified based on the LDH released into the medium, as described above. We found that SZ stimuli-induced cell death was also significantly increased by KLHDC10 knockdown in RAW264.7 cells (Fig 4C and 4D). In addition, as cell death was completely abrogated with Necrostatin-1 treatment, we confirmed that SZ stimuli-induced cell death was RIP1/3 kinases-dependent necroptosis (Fig 4C and 4D).

In fibroblastic cell lines, such as immortalized MEF cells and L929 cells, RIP1/3-dependent necroptosis was also induced by SZ stimulation. Intriguingly, however, the KLHDC10 deficiency-induced enhancement of cell death could not be observed (Fig 4E to 4H). These results did not conflict with the *in vivo* observation that systemic necroptosis was comparable between WT and KLHDC10 KO mice (Fig 2A and 2B). These results suggest that KLHDC10 deficiency-induced enhancement of RIP1/3-dependent necroptosis occurs selectively in inflammatory cells.

## Discussion

In this study, we showed that KLHDC10 KO mice are resistant to TNFα-induced SIRS. Recent reports have revealed that necroptosis and the subsequent release of excessive inflammatory cytokines play central roles in TNFα-induced SIRS. We found that KLHDC10 deficiency did not affect systemic necroptosis (Fig 2A and 2B), whereas it significantly reduced the induction of IL-6 mRNA expression in the spleen and IL-6 release into the serum (Fig 2D and 2C, respectively).

Inflammatory cytokines play important roles in pathogen defense [37]. However, excessive inflammatory cytokine release may cause some damage on individuals, as observed, for example, in inflammatory bowel disease and brain infarction [38,39,40]. In TNFα-induced SIRS, excessive IL-1β and IL-6 production has been shown to play a role in regulating survival. In particular, pretreatment with IL-1 receptor antagonists or IL-6 antibodies was reported to rescue mortality in TNFα-induced SIRS [23,24]. Moreover, IL-17 receptor deletion or pretreatment with IL-17 antibodies, as well as IFN-α or IFN-β deletion, protected mice from excessive IL-6 release into serum, hypothermia, and survival [28,41]. Therefore, the diminished inflammatory responses in KLHDC10 KO mice may contribute to resistance against TNFα-induced SIRS. Further, subsequent *in vitro* analysis revealed that KLHDC10 deficiency selectively promoted RIP1/3-dependent necroptosis in inflammatory cells (Fig 4C to 4H). Therefore, we can speculate that the enhanced cell death of inflammatory cells might contribute to the reduced inflammatory responses in KLHDC10 KO mice (Fig 4I).

A significant reduction of IL-6 mRNA expression in KLHDC10-deficient mice was observed in the spleen (Fig 2D). The spleen is known to be one of the organs in which acute inflammation is observed in SIRS patients. In contrast, in the liver of KLHDC10 KO mice, no significant reduction of IL-6 mRNA was observed (Fig 2D). The precise reason for this tissue specificity is currently unknown. Because enhanced necroptosis in the presence of KLHDC10 deficiency was selectively observed in inflammatory cells (Fig 4C to 4H), one possible explanation might be the difference in the proportion of inflammatory cells to other cell types, including parenchymal cells. Thus, we hypothesize that the proportion of inflammatory cells in other tissues, such as the liver, is too small to allow us to detect the effects of KLHDC10 deficiency upon IL-6 mRNA induction when the whole organ was analyzed.

Pre-treatment with Necrostatin-1 suppressed the enhanced cell death observed in KLHDC10-deficient inflammatory cells, suggesting that KLHDC10 acts as a cell type-specific regulator of RIP1/3 kinases-dependent necroptosis. The reason why the effects of KLHDC10 deficiency on cell death were only observed in inflammatory cells remains elusive. It has been reported that NADPH oxidase 1 (Nox1)-mediated superoxide production is involved in necroptosis under the stimulation of TNFα in fibroblastic cell lines, including L929 cells [14]. In contrast, it is known that in phagocytes, such as macrophages and neutrophils, NADPH oxidase 2 (Nox2) contributes to superoxide production [15,16,17]. Nox2-mediated superoxide production is implicated in the killing of bacteria through opsonization by phagocytic cells. We revealed that Nox2 knockdown partially suppressed SZ stimuli-induced necroptosis (S3A Fig), suggesting that Nox2 is also involved in necroptosis induction. In addition, the enhancement of necroptosis by KLHDC10 knockdown was partially but significantly restored by double knockdown of KLHDC10 and Nox2 (S3B Fig). Notably, Nox2 is known to be specifically expressed in phagocytes [14,42]. Considering these data, one conceivable explanation for the cell type-specific involvement of KLHDC10 in necroptosis could be attributed to the difference in the signaling components that mediate necroptosis, including Nox2, between inflammatory cells and fibroblastic cells.

One appealing follow-up of this study is the investigation of the molecular targets of KLHDC10 that are engaged in necroptosis in inflammatory cells. Because ASK1 KO mice were not protected from mortality and hypothermia in TNFα-induced SIRS (Fig 1A to 1C), enhanced necroptosis with KLHDC10 deficiency may be related to other molecules rather than ASK1. Our previous studies suggest that KLHDC10 may have two different functions, i.e., the suppression of PP5 and the activity as substrate receptor of the CRL2 complex. In future studies, we will need to clarify which function of KLHDC10 is involved in the regulation of necroptosis.

Besides, it has been widely accepted that necroptosis triggers inflammatory responses, and thus aggravates SIRS pathogenesis. In this respect, our model indicated in Fig 4I will provide a possibility that necroptosis not only exacerbates the pathology of SIRS but also weakens the severe inflammatory responses in SIRS, depending on the kind of cells dying from necroptosis. However, in order to confirm its validity, it is highly expected to show that KLHDC10 deficiency enhances necroptosis of inflammatory cells *in vivo* in future studies. At the same time, we do not exclude the possibility that the other mechanisms may also be involved in the resistance of KLHDC10 KO mice against SIRS model.

At present, there is no effective therapeutic strategy available for the treatment of SIRS. We hope that an advanced understanding of KLHDC10 functions in TNFα-induced SIRS will provide attractive targets for the cure of this severe and acute illness.

## Supporting Information

S1 Fig Generation of KLHDC10 knockout (KO) mice. (A) A schematic diagram of the targeting vector and the targeted allele of the *KLHDC10* gene. Exon 1, containing the ATG codon, was replaced with a neomycin-resistant gene cassette. The 5’probe or 3’probe used for Southern blotting is indicated as a blue or green bold line, respectively. S, ScaI restriction site; B, BamHI restriction site. PKGp, phosphoglycerate kinase 1 promoter; neo^r^, neomycin-resistant gene cassette; tk, thymidine kinase. (B) Southern blot to confirm the mutant allele integration. Genomic DNAs were digested using the indicated restriction enzymes and hybridized with specific probes.
(C) Absence of KLHDC10 expression was confirmed by immunoblotting with the KLHDC10 antibody (S-1). Lysate of MEFs derived from WT mice and KLHDC10 KO mice were used.
(PDF)

S2 Fig KLHDC10 deficiency does not enhance TNFα plus Z-VAD-fmk-induced necroptosis in inflammatory cells *in vitro*. (A) RAW264.7 cells transfected with control or mKLHDC10 siRNAs were stimulated as indicated. After 24 hours, LDH release was quantified as an indicator of cell death (n = 4). Data are represented as the mean ± SEM. T: mTNFα (20 ng/ml), Z: Z-VAD-fmk (10 μM).
(PDF)

S3 Fig Nox2 is partially involved in the enhanced necroptosis induced by KLHDC10 deficiency in inflammatory cells. (A, C) RAW264.7 cells transfected with control siRNA, mKLHDC10, and mNox2 siRNAs were stimulated as indicated. After 24 hours, LDH release was quantified as an indicator of cell death (n = 3 for A, n = 5 for C). (B, D) The knockdown efficiency of KLHDC10 or Nox2 was determined by immunoblotting analysis after transfection of RAW264.7 cells with the indicated siRNAs. Data are represented as the mean ± SEM. *P<0.05, **P<0.01 analyzed using a one-way ANOVA with Dunnette’s post-hoc test.
(PDF)
