# Supplementary figures and images for: KLHDC10 Deficiency Protects Mice against TNFα-Induced Systemic Inflammation
Source: PLoS One. 2016 Sep 15;11(9):e0163118. doi: 10.1371/journal.pone.0163118 (PMC5025154; doi:10.1371/journal.pone.0163118)

Sup. Figure 1

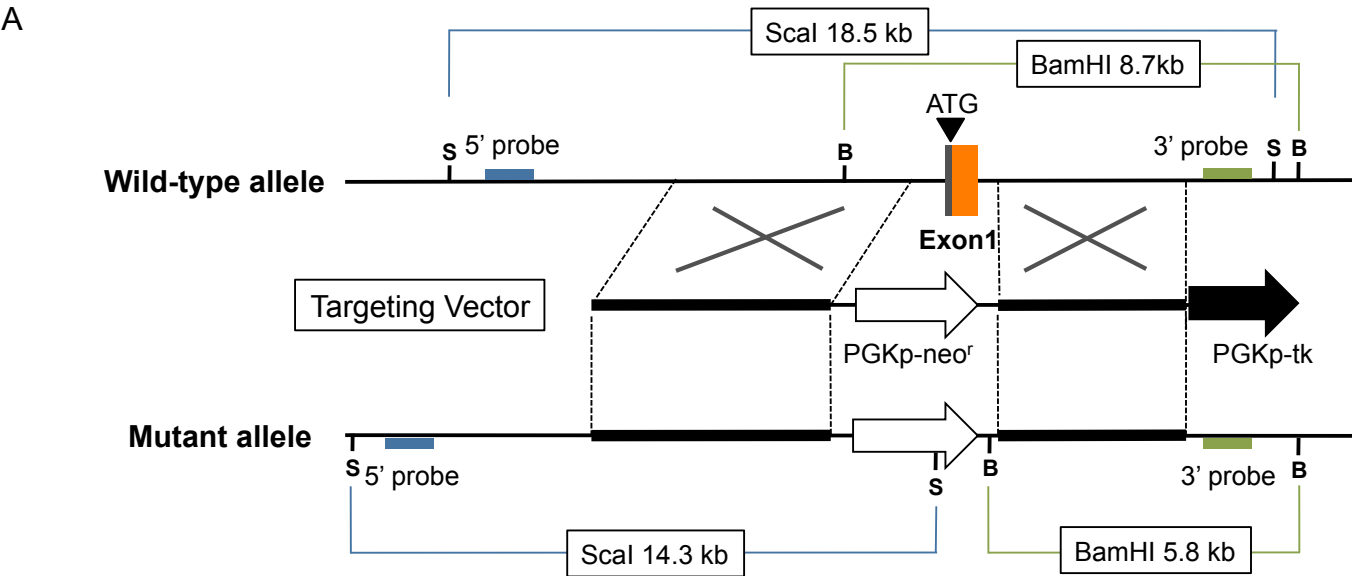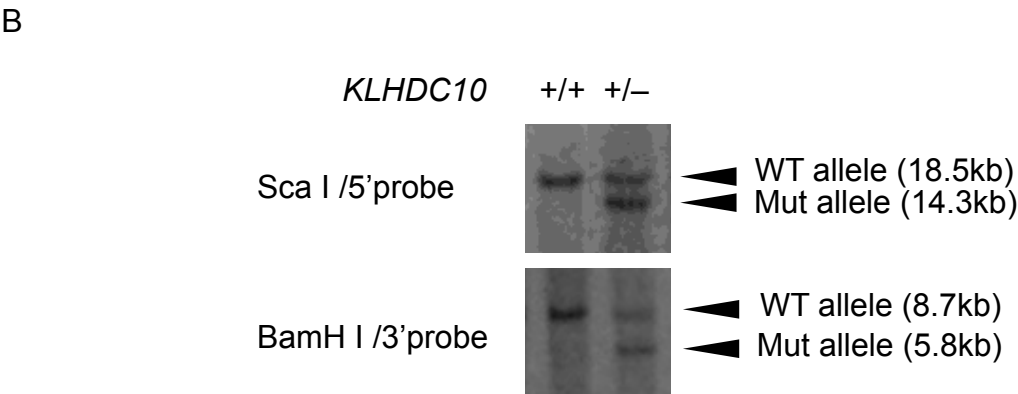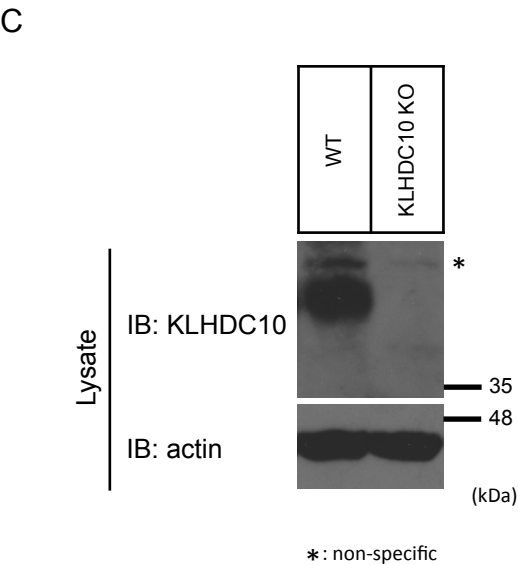

Supplement: S1 Fig — (A) A schematic diagram of the targeting vector and the targeted allele of the KLHDC10 gene. Exon 1, containing the ATG codon, was replaced with a neomycin-resistant gene cassette. The 5’probe or 3’probe used for Southern blotting is indicated as a blue or green bold line, respectively. S, ScaI restriction site; B, BamHI restriction site. PKGp, phosphoglycerate kinase 1 promoter; neor, neomycin-resistant gene cassette; tk, thymidine kinase. (B) Southern blot to confirm the mutant allele integration. Genomic DNAs were digested using the indicated restriction enzymes and hybridized with specific probes. (C) Absence of KLHDC10 expression was confirmed by immunoblotting with the KLHDC10 antibody (S-1). Lysate of MEFs derived from WT mice and KLHDC10 KO mice were used. (PDF) [file pone.0163118.s001.pdf]

Sup. Figure 2

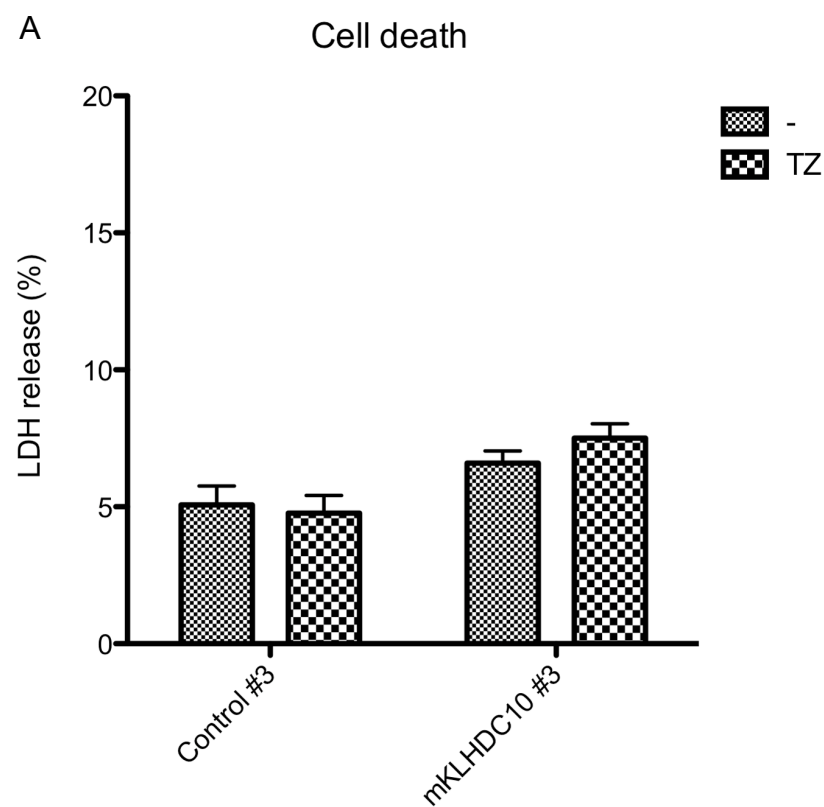

Supplement: S2 Fig — (A) RAW264.7 cells transfected with control or mKLHDC10 siRNAs were stimulated as indicated. After 24 hours, LDH release was quantified as an indicator of cell death (n = 4). Data are represented as the mean ± SEM. T: mTNFα (20 ng/ml), Z: Z-VAD-fmk (10 μM). (PDF) [file pone.0163118.s002.pdf]

Sup. Figure 3

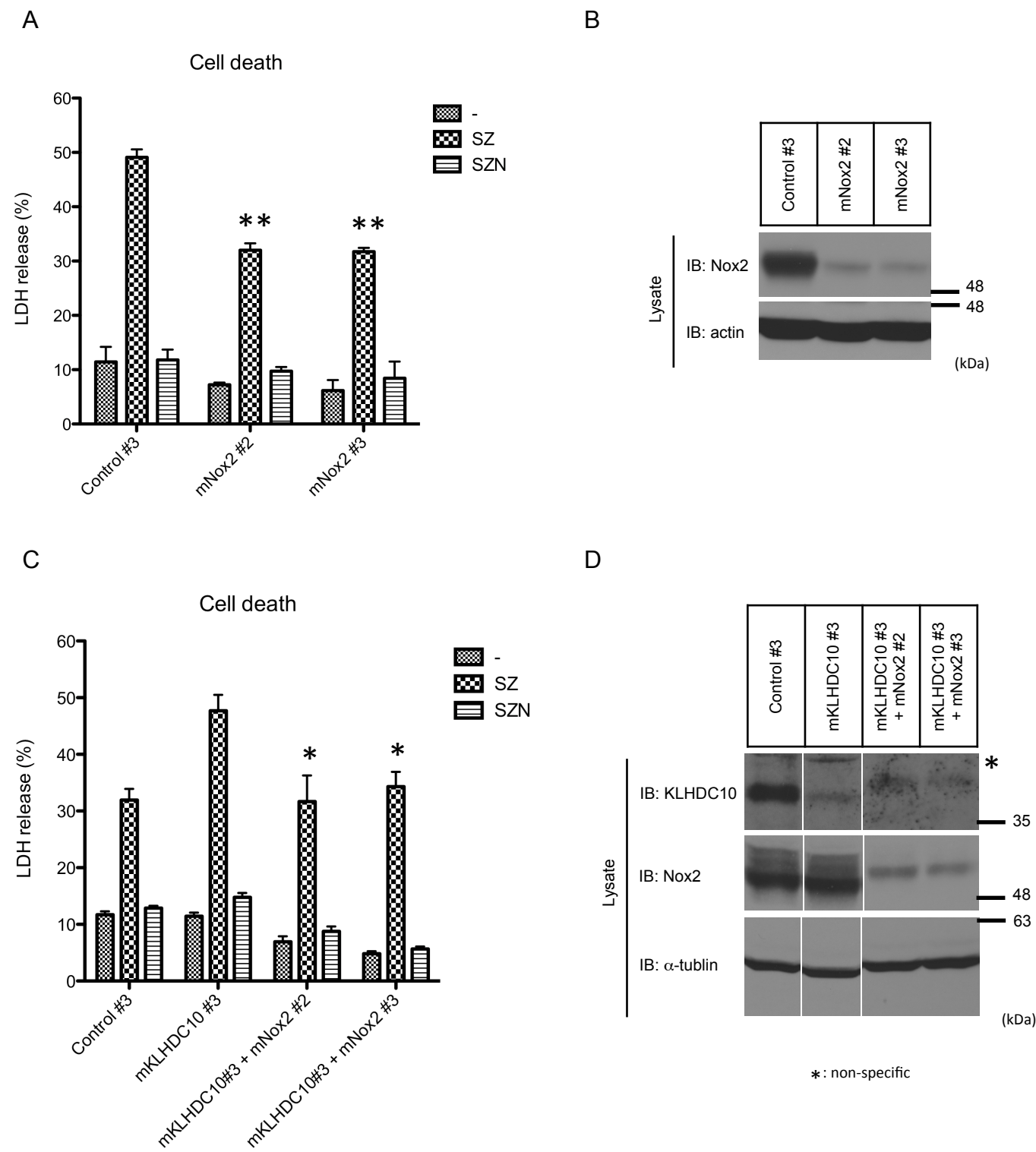

Supplement: S3 Fig — (A, C) RAW264.7 cells transfected with control siRNA, mKLHDC10, and mNox2 siRNAs were stimulated as indicated. After 24 hours, LDH release was quantified as an indicator of cell death (n = 3 for A, n = 5 for C). (B, D) The knockdown efficiency of KLHDC10 or Nox2 was determined by immunoblotting analysis after transfection of RAW264.7 cells with the indicated siRNAs. Data are represented as the mean ± SEM. *P<0.05, **P<0.01 analyzed using a one-way ANOVA with Dunnette’s post-hoc test. (PDF) [file pone.0163118.s003.pdf]
